# How crime data incompleteness shapes ethnic disparities in predictive policing: evidence from Chicago

**DOI:** 10.1186/s40163-026-00306-9

**Published:** 2026-10-14

**Authors:** Mengqin K. Jiang, Federico Liberatore, Alun Preece

**Affiliations:** School of Computational and Mathematical Sciences, Cardiff University, Senghennydd Road, Cardiff, CF24 4AG UK

**Keywords:** Predictive policing, Data incompleteness, Enforcement bias, Fairness

## Abstract

**Purpose:**

Fairness in Predictive Policing (PP) has been explored by many researchers. However, to the best of our knowledge, no work has quantified the impact of event-level data incompleteness in spatio-temporal prediction on each ethnicity. In this paper, we propose a model to quantify the impact of recorded crime data incompleteness on ethnic disparities in policing. We also examine whether these disparities depend on where in the city data are missing.

**Methods:**

We introduce a neighbourhood-level method that allocates PP prediction errors (false positives as potential over-policing, false negatives as potential under-policing) to ethnic groups in proportion to their share of the neighbourhood population. Using Chicago crime records (2022–2024), census data, and HAGEN (a state-of-the-art spatial-temporal PP model), we explore data incompleteness by reducing the original crime data at varying ratios in two settings: (i) 25–100% reductions in neighbourhoods with low racial diversity; (ii) 50% or 100% reductions in communities dominated by a single ethnicity (Hispanic, Non-Hispanic Black, Non-Hispanic White, or Non-Hispanic Asian). Enforcement bias outcomes are then compared across ethnicities and scenarios.

**Results:**

Removing records from a single-ethnicity-majority community generally reduces predicted over-policing and increases predicted under-policing for that ethnicity across the whole study area, with Asian-majority scenarios a consistent exception. In low-diversity neighbourhoods, greater missingness amplifies prediction bias against Non-Hispanic Black residents specifically: predicted over-policing falls and predicted under-policing rises, with the opposite holding for other groups. The largest overall shifts occur when data are removed from Black-majority communities.

**Conclusions:**

Incomplete crime records could propagate into substantial, ethnically unequal disparities in model-predicted policing exposure, whose direction depends on where missingness concentrates. The findings motivate auditing missingness and its demographic distribution as a prerequisite for deploying event-level PP systems.

## Introduction

### Background

The United Kingdom (UK) is planning to use Artificial Intelligence (AI) to prevent crime by predicting criminal activity and allocating police resources according to forecasting results before 2030 (Department for Science, 2025). However, the technology used in crime mapping can also be risky because some communities might be unfairly profiled. For example, in the US, reports in 2023 noted that AI-powered facial recognition software failed to identify Black faces accurately (Bbc News, 2024), increasing concerns about bias affecting some ethnic groups. More broadly, the data used for prediction can also reflect social and institutional processes (e.g., reporting, detection, and recording practices), so biases in crime records may propagate into downstream policing decisions.

Predictive policing (PP) uses historical crime records to forecast where and when future events may occur (Meijer & Wessels, 2019). Using the information provided by these predictions, police agencies may deploy policing resources accordingly. In practice, a key problem is *data incompleteness*. Crime records are often missing events. Data missingness can result from under-reporting, uneven detection, and administrative recording choices (Spence & Crivatu, 2025). It is therefore unlikely to be random. It can also vary across neighbourhoods and across ethnic groups. Prior work shows that missing data can reduce PP accuracy (Strikwerda, 2021). There is also broader evidence that AI tools in crime-related settings may reflect or reinforce bias (Joseph, 2025).

### Literature review

Predictive Policing (PP) aims to forecast crime and support police enforcement (Meijer & Wessels, 2019). This section reviews (i) event-level spatio-temporal PP models, (ii) crime data incompleteness and under-reporting, and (iii) how biased data and prediction errors can translate into enforcement bias, with particular attention to disparities across groups.

Many papers have been published to predict crime not only geographically but also temporally. By considering temporal recurrence and spatial dependency simultaneously within and across regions, Sun et al. (2020) proposed a spatio-temporal framework, CrimeForecaster, which was reported to improve performance by up to 21% compared to other popular algorithms at the time. Building upon CrimeForecaster, Wang et al. (2022) proposed the HAGEN (Homophily-Aware Graph Convolutional Recurrent Network) to capture crime relationships between areas that have similar crime patterns despite being far apart. Their experiments show that HAGEN outperforms prior methods. Considering correlations among multiple crime types, Wang et al. (2025) introduced MRAGNN (Multi-type Relations Aware Graph Neural Networks) to predict crime occurrences, and report improvements over several existing techniques. More recently, Albors Zumel et al. (2025) developed a ConvLSTM deep learning framework for event-level spatio-temporal crime forecasting across four US cities including Chicago, finding that integrating mobility and sociodemographic data improves predictive performance, particularly at fine spatial and temporal resolutions. Field evaluations of PP deployment further complicate the picture: Galiani and Jaitman (2023) found no statistically significant crime-reduction benefit from a US-developed PP system over local crime analysts in a randomised controlled trial in Montevideo, Uruguay, underscoring that predictive accuracy alone does not guarantee operational effectiveness. In this paper, we focus on *spatial-temporal* prediction with event-level data, i.e., forecasting crime occurrences in space and time. This differs from *hotspot* prediction that only ranks or flags high-risk areas without predicting specific events.

Considering bias in historical data (Richardson et al., 2019), algorithms (Babuta & Oswald, 2019), and enforcement (Rotaru et al., 2022), fairness in PP has been studied by a number of researchers (Alikhademi et al., 2022; Arowosegbe, 2023; Babuta & Oswald, 2019). Almasoud and Idowu (2025) provide a systematic review of algorithmic fairness in PP and find that most existing work focuses on racial bias while age, gender, and socio-economic status receive less attention; they also highlight that police enforcement actions can themselves introduce discriminatory feedback loops into recorded crime data, reinforcing model bias in subsequent prediction cycles.

Data sources used in PP have been controversial for many years (Alikhademi et al., 2022). Common issues such as operational bias in recording crimes and under-reported crimes can lead to biased data (Akpinar et al., 2021; Brantingham, 2017). With biased data, forecasting results can be inaccurate, leading to flawed predictions (Richardson et al., 2019); indeed, empirical evidence suggests that official arrest records are themselves racially biased measures of individual criminal behaviour (Grunwald, 2024). Therefore, studying biased data is important in PP.

Currently, the completeness of crime data is widely assessed and studied by researchers in policing and criminal justice systems. Incompleteness may arise from missing records, under-reporting, and data-quality issues, and these processes can vary across neighbourhoods and communities, and they may correlate with ethnicity and other demographic factors. Evidence of missingness appears in multiple data sources. In 2008, Lynch and Jarvis (2008) analysed the distribution of missing crime data in the Uniform Crime Reporting (UCR) program, a major data source in the US since 1929. Their research indicated that data incompleteness is widespread nationally. Later, Lageson (2023) highlighted the spread of inaccurate criminal records and the societal harms of bad data for fairness, proposing several data-management solutions for criminal legal systems. In the same year, Xie et al. (2023) investigated National Crime Victimization Survey (NCVS) data and found that victims’ willingness to report crimes has decreased, while mistrust of police has increased, since the early 21st century.

Considering event-level missing information, Buil-Gil et al. (2022) studied the likelihood of unrecorded crimes and found that, compared with larger geographical areas, micro-level crime analysis may be more vulnerable to bias. Targeting under-reported crimes, Riascos Villegas et al. (2023) proposed a spatio-temporal events model. Validation using crime data from Bogotá (Colombia) indicates reliability in estimating under-reporting rates.

In addition, individual-level incompleteness can influence the fairness of PP. Given missingness patterns in crime data, Udoh (2020) explored the impact of incomplete crime-record patterns on predicted results, finding that poor-quality crime records lead to biased predictions. Outside the policing domain, Teeple et al. (2023) demonstrated that missing data in electronic health records can affect model predictive performance differently across racial groups, finding that reducing missingness produced modest but significant changes in group-specific metrics such as false positive and false negative rates; this supports treating data incompleteness as a fairness concern in its own right rather than a purely technical issue.

In the criminal justice domain specifically, Stockton et al. (2024) demonstrated through simulation that even less than 10% of missing data can produce biased estimates of racial disparities in sentencing when missingness is non-random, and that the direction of bias depends on the missingness pattern, reinforcing the case for treating incompleteness as a substantive fairness concern rather than a data-cleaning step.

Using the Crime Survey for England and Wales, Brunton-Smith et al. (2023) generated synthetic census and crime data to estimate victimisation and reporting willingness for each population unit. Using these synthetic data, they estimate spatial undercounting and argue their method has the potential to correct existing strategies.

Concerning biased and incomplete data, existing papers on PP focus mainly on improving predictive models. Wu et al. (2020) proposed a Bayesian hierarchical model to improve the fairness and accuracy of crime predictions, focusing on under-reported crimes when using mobility-based models. To reduce bias in incomplete tabular data, Miao et al. (2025) introduced a Transformer-based fairness-aware prediction model (FATE) to improve algorithmic fairness and predictive accuracy. Combining domain knowledge and in-processing methods, Wu and Frias-Martinez (2024) introduced a deep learning framework to enhance predictions; however, their results indicate improved fairness at the cost of reduced accuracy.

Biased forecasting results can lead to biased policing decisions (Akpinar et al., 2021; Ziosi & Pruss, 2024). Rotaru et al. (2022) forecast crime in Chicago and highlighted uncertainty in crime patterns, indicating that disadvantaged socio-economic status is associated with increased enforcement bias; however, only socio-economic factors were discussed in that paper. More generally, when prediction errors and data incompleteness differ across neighbourhoods, the resulting over- and under-policing can also differ across communities.

In a closely related Chicago study, Piza et al. (2025) found that gunshot detection technology produced racially disparate enforcement outcomes relative to conventional calls-for-service responses, highlighting that technology-assisted policing can amplify group disparities independent of underlying crime rates.

Considering the racial context in the US, Jackson et al. (2023) investigated racism and enforcement bias in Black communities and found that over-policing and under-policing are strongly related to perceived police racism. The research in Jones-Brown and Williams (2021) also reveals enforcement bias against Black communities. Taking crime characteristics, including individual-level data, into account, Fogliato et al. (2024) presented a parametric regression-based two-step estimator to model the likelihood of crime reporting and arrest. After adjusting for unreported crimes, the probability of arrest drops significantly and racial disparities among offenders become smaller. Not limited to race, to reduce enforcement bias caused by non-reporting crimes in vulnerable communities, Das et al. (2025) introduced a Wasserstein GAN (WGAN)-driven likelihood-free approach; however, that work focuses on forecasting crime hotspots.

In summary, unreported crimes and enforcement bias have been explored in many papers. By analysing under-reporting, several works propose models to improve predictive fairness. Since predictive inaccuracy and biased data can lead to enforcement bias, racial and socio-economic biases have been highlighted by multiple researchers. However, the literature mainly studies either individual-level data, or event-level data focused on hotspot prediction, rather than examining how event-level data incompleteness affects spatio-temporal PP predictions and associated over- and under-policing disparities across ethnicities at the neighbourhood level. To the best of our knowledge, no prior work quantifies these impacts under controlled missingness scenarios across different demographic contexts, which motivates the focus of this paper.

### Research questions and paper outlines

By reviewing the related literature, we found that numerous papers have discussed algorithmic bias and proposed several methods to improve robustness and fairness (Almasoud & Idowu, 2025)(Alikhademi et al., 2022). However, less is known about how spatial-temporal PP models with event-level data behave when there is substantial missing data, particularly when missingness is uneven across communities and may encode enforcement and reporting biases. The aim of this paper is to explore how incomplete crime data can translate into *enforcement bias* across ethnicities as, in practice, PP results support policing deployment and enforcement. In this work, we use *enforcement bias* to refer to group disparities in predicted policing exposure induced by model errors; specifically, differences in over- and under-prediction (which may lead to over- and under- policing, respectively) when comparing predicted events to recorded events at the neighbourhood level. Throughout the paper, over-policing and under-policing therefore denote potential policing exposure derived from model predictions, and not observed police behaviour.

Therefore, the research questions of this paper are the following:

- $RQ_\textrm{1}$: As there are always gaps between recorded crimes and crimes predicted by PP models, how to quantify potential disparities in policing exposure across ethnic groups arising from these differences?
- $RQ_\textrm{2}$: How do policing policies derived from PP models trained on incomplete data influence outcomes across ethnic groups?

To answer these questions, we make three main contributions. First, we introduce a neighbourhood-level method to quantify enforcement bias by ethnicity from spatio-temporal prediction errors. Second, to evaluate the method and explore the impact of missing information on PP forecasting, we design and implement controlled data-reduction experiments. Third, we apply both to a real case study. To exemplify our methodology, we use Chicago crime data from 2022 to 2024 and the state-of-the-art (SOTA) spatial-temporal PP model HAGEN (Wang et al., 2022). We vary the degree of missingness under different demographic contexts, including low-diversity neighbourhoods and single-ethnicity-majority communities. We then measure how over- and under-prediction disparities change across ethnic groups. This analysis clarifies how incomplete records can propagate into enforcement bias, and highlights the importance of auditing missingness and its demographic distribution before deploying PP systems.

The rest of this paper is organised as follows. Section “Methodology” introduces the methodology of this paper, including the problem definition and the model to quantify bias on each ethnicity. To verify the reliability of the proposed methodology, a case study is presented in Section “Case study”. Finally, Section “Discussion and conclusions” discusses the results, summarises the conclusions, and states the limitations of this work.

## Methodology

To address the research questions, this work introduces a methodology to quantify enforcement bias for each ethnicity.

### Problem definition

Following the research questions in Section “Introduction”, this section introduces the problem definitions. The notation used in the definitions is summarised in Table 1.

**Table 1:** Summary of notations in this paper

*Sets*	
*N*	Set of neighbourhoods
*C*	Set of crime types
*E*	Set of ethnic groups
$Population_n$	Total population of neighbourhood *n*
$Proportion_{ne}$	Proportion of ethnicity *e* in neighbourhood *n*
$T_M$	Set of time slots in a sequence of length *m*
*Indices*	
*n ∈ N*	Neighbourhood area
*c ∈ C*	Crime type
*e ∈ E*	Ethnic group
$t_m \in T_M$	Time slot in a sequence of length *m*
*Variables*	
$A_{nc}^{t_m}$	Actual occurrence of crime type *c* in neighbourhood *n* at time $t_m$
$P_{nc}^{t_m}$	Predicted occurrence of crime type *c* in neighbourhood *n* at time $t_m$
$PE_{nc}^{t_m}$	Prediction error of crime *c* in neighbourhood *n* at time $t_m$
$FP_{nce}^{t_m}$	Indicator of over-prediction impact (i.e., potential over-policing) for ethnicity *e* in neighbourhood *n* for crime *c* at time $t_m$
$FN_{nce}^{t_m}$	Indicator of under-prediction impact (i.e., potential under-policing) for ethnicity *e* in neighbourhood *n* for crime *c* at time $t_m$
$ProportionFP_{ce}$	Proportion of over-prediction for ethnicity *e* within crime type *c*
$ProportionFN_{ce}$	Proportion of under-prediction for ethnicity *e* within crime type *c*
$SDI_n$	Simpson’s Diversity Index of neighbourhood *n*

- *Definition 1: Crime records* We follow the definition of crime records proposed by Wang et al. (2022). In a time window *T*, crimes occur in non-overlapping and consecutive time slots $T=(t_1,\ldots ,t_m)$, where *m* is the sequence length. In neighbourhood *n*, $A_{nc}^{t_m}$ denotes the actual (recorded) value of crime type *c* in time slot $t_m$. In this paper, crimes are represented as binary variables: we set $A_{nc}^{t_m}=1$ if crime type *c* occurs in area *n* during time slot $t_m$, and 0 otherwise. Therefore, the predicted value $P_{nc}^{t_m}$ is also binary.
- *Definition 2: Geographical neighbourhoods* We adopt the same neighbourhood definition as Wang et al. (2022). We represent a city with *N* neighbourhoods as a weighted *directed* graph $\varsigma =(\nu ,\xi ,\omega )$, where *ν * is the set of nodes and *|ν |=N*. *ξ * is the set of directed edges between region nodes. *ω * is the weighted adjacency matrix, where $\omega \in \mathbb {R}^{N \times N}$. $\omega _{i,j}$ denotes the strength of influence from region *i* to region *j*.
- *Definition 3: Racial diversity* This paper uses Simpson’s Diversity Index (SDI) to measure racial diversity in a neighbourhood (McLaughlin et al., 2016; Nguyen, 2017). The SDI of neighbourhood *n* is calculated as:
  1 $$\begin{aligned} SDI_n = 1 - \sum _{e}^{E} Proportion^2_{ne} \end{aligned}$$
- *Definition 4: Majority of the community* If the proportion of an ethnicity *e* in a neighbourhood is both the largest and greater than 50%, we define that neighbourhood as *e*-majority (or *e*-major).
- *Definition 5: Enforcement bias* Enforcement bias in a neighbourhood relates to potential over-policing and under-policing. PP predictions are often used to prioritise patrols and allocate attention across neighbourhoods (Meijer & Wessels, 2019). As discussed in Section “Literature review”, biased or incomplete crime records can distort forecasts (Akpinar et al., 2021; Brantingham, 2017; Richardson et al., 2019) and propagate into biased enforcement decisions (Akpinar et al., 2021; Ziosi & Pruss, 2024). We therefore interpret prediction errors as proxies for misallocated policing exposure: false positives (predicting a crime when none is recorded) correspond to over-prediction, which may lead to over-policing; false negatives (missing a recorded crime) correspond to under-prediction, which may lead to under-policing. This allows us to quantify enforcement-bias disparities across ethnic groups.^1^

### Quantifying enforcement bias

In this section, we introduce a model to quantify enforcement bias of spatio-temporal prediction models for each ethnicity.

Given the actual occurrence values $A_{nc}^{t_m}$ and predictions $P_{nc}^{t_m}$, the prediction error can be calculated as:

2 $$\begin{aligned} PE_{nc}^{t_m} = A_{nc}^{t_m} - P_{nc}^{t_m} \end{aligned}$$

As $A_{nc}^{t_m}$ and $P_{nc}^{t_m}$ are binary, $PE_{nc}^{t_m} \in \{-1,0,1\}$. Following Definition 5, we characterise outcomes as:

3 $$\begin{aligned} PE_{nc}^{t_m} = {\left\{ \begin{array}{ll} 0 & \text {Accurate prediction} \\ 1 & \text {Under-prediction (false negative, potential under-policing)} \\ -1 & \text {Over-prediction (false positive, potential over-policing)} \\ \end{array}\right. } \end{aligned}$$

Accordingly, we define the following indicators for each ethnicity *e* in neighbourhood *n*:

4 $$\begin{aligned} FP_{nce}^{t_m} = {\left\{ \begin{array}{ll} 1 & \text {if } PE_{nc}^{t_m}=-1\\ 0 & \text {otherwise,} \end{array}\right. } \qquad FN_{nce}^{t_m} = {\left\{ \begin{array}{ll} 1 & \text {if } PE_{nc}^{t_m}=1\\ 0 & \text {otherwise.} \end{array}\right. } \end{aligned}$$

#### Over-prediction

The impact of over-prediction for crime type *c* on ethnicity *e* across all neighbourhoods is:

5 $$\begin{aligned} FP_{ce} = \sum _{t_m \in T_M}\sum _{n \in N} FP_{nce}^{t_m} \cdot Population_n \cdot Proportion_{ne}. \end{aligned}$$

Its proportion is:

6 $$\begin{aligned} ProportionFP_{ce} = \frac{FP_{ce}}{\sum _{e^\prime \in E} FP_{ce^\prime }}. \end{aligned}$$

#### Under-prediction

Similarly, the impact of under-prediction for crime type *c* on ethnicity *e* across all neighbourhoods is:

7 $$\begin{aligned} FN_{ce} = \sum _{t_m \in T_M}\sum _{n \in N} FN_{nce}^{t_m} \cdot Population_n \cdot Proportion_{ne}. \end{aligned}$$

Its proportion is:

8 $$\begin{aligned} ProportionFN_{ce} = \frac{FN_{ce}}{\sum _{e^\prime \in E} FN_{ce^\prime }}. \end{aligned}$$

We allocate false positives and false negatives to ethnic groups in proportion to each group’s share of the neighbourhood population (Eqs. 5 and 7). That is, we use neighbourhood demographic composition as a proxy for the impact that police operations have on individuals of each ethnicity.^2^

## Case study

Given the availability of crime and demographic data, the proposed methodology in Section “Methodology” is applied to Chicago. Chicago is the largest city in Illinois (US) and covers approximately 607.44 $\text {km}^2$. As of 2020, it has about 2,746,388 residents and a population density of 4656.33/$\text {km}^2$. Geographically, Chicago is divided into 77 community areas (City Of, 2024).

We use the Chicago case study to exemplify the proposed methodology rather than to draw definitive conclusions about policing in Chicago. The published records are themselves known to be incomplete and should not be taken as ground truth (Grunwald, 2024); we adopt them as the baseline input that would be available to a PP system, and examine how further, induced missingness propagates through PP forecasts and, potentially, into the policing operations such forecasts would inform.

### Dataset

The recorded crime data used in this paper are obtained from the Chicago Data Portal (City Of, 2026). They include crime ID numbers and the corresponding crime types, locations, community areas, and times. Demographic distribution data for Chicago are extracted from Illinois Action for Children (Illinois Action for Children, 2026),^3^ including the population and the proportion of each ethnicity in every community area. According to the collected data, ethnic groups in Chicago are classified as: Hispanic, Non-Hispanic Black, Non-Hispanic White, Non-Hispanic Asian, and Non-Hispanic Other or Multiple Races.

Given the influence of Covid-19, only 2022–2024 crime data are used. Eight crime types are considered in this study: theft, criminal damage, narcotics, robbery, assault, deceptive practices, burglary, and battery.^4^

As defined in Section “Problem definition”, there are nine low racial diversity communities (i.e., *SDI ≤ 0.15*), 18 Hispanic-major communities, 28 Non-Hispanic Black-major communities, 19 Non-Hispanic White-major communities, and one Non-Hispanic Asian-major community.

### Experimental assumptions

To explore the research questions, this paper proposes the following assumptions:

*Datasets:* This paper uses recorded crime data from the Chicago Data Portal (City Of, 2026) because they are the most comprehensive public, city-wide source available and a standard input in predictive policing research. Therefore, we use these records as the baseline of this paper. For the purposes of this case study only, we treat the recorded crime data as the reference against which predictions are evaluated, i.e., as if they represented the crimes that occurred. This is an operational assumption of the experiments and not a general claim: recorded crimes are not occurred crimes. A large share of crime is never reported, and the willingness to report varies across crime types, communities, and levels of trust in the police (Buil-Gil et al., 2022; Spence & Crivatu, 2025; Xie et al., 2023). Recorded data also reflect detection and recording practice, so crime and arrest records are themselves biased measures of criminal behaviour (Fogliato et al., 2024; Grunwald, 2024). We adopt the assumption because no ground truth of occurred crimes is available at the community-area level, and because these records are the input that a deployed PP system would use. All our results must therefore be read as *relative* changes with respect to this already incomplete baseline, as discussed in Sections “Case study” and Section “Limitations and Future Work”.

*Model:* We assume the police will use the PP models with best performance in accuracy and robustness. HAGEN is a supervised learning algorithm proposed by Wang et al. (2022). As reported by Wang et al. (2025) and Wang et al. (2022), HAGEN demonstrates strong performance and robustness when compared with nine other models in precision, recall, Micro-F1, Macro-F1, ROC_AUC, and training/validation/testing time, including both traditional and SOTA models. Therefore, HAGEN is used as the benchmark PP model in this paper. It is important to highlight that HAGEN predicts crime occurrences in predefined regions within a given time slot; in particular, when any crime occurs in the period, the value is recorded as 1, regardless of the number of occurrences.

*Enforcement bias:* Following the definition of PP proposed by Meijer and Wessels (2019), we assume that policing strategies are informed by the prediction results of PP. Thus, this paper uses prediction errors as a proxy for enforcement bias, where false positives (FP) may lead to over-policing and false negatives (FN) may lead to under-policing in the corresponding neighbourhoods.

### Experimental design

**Fig. 1:**
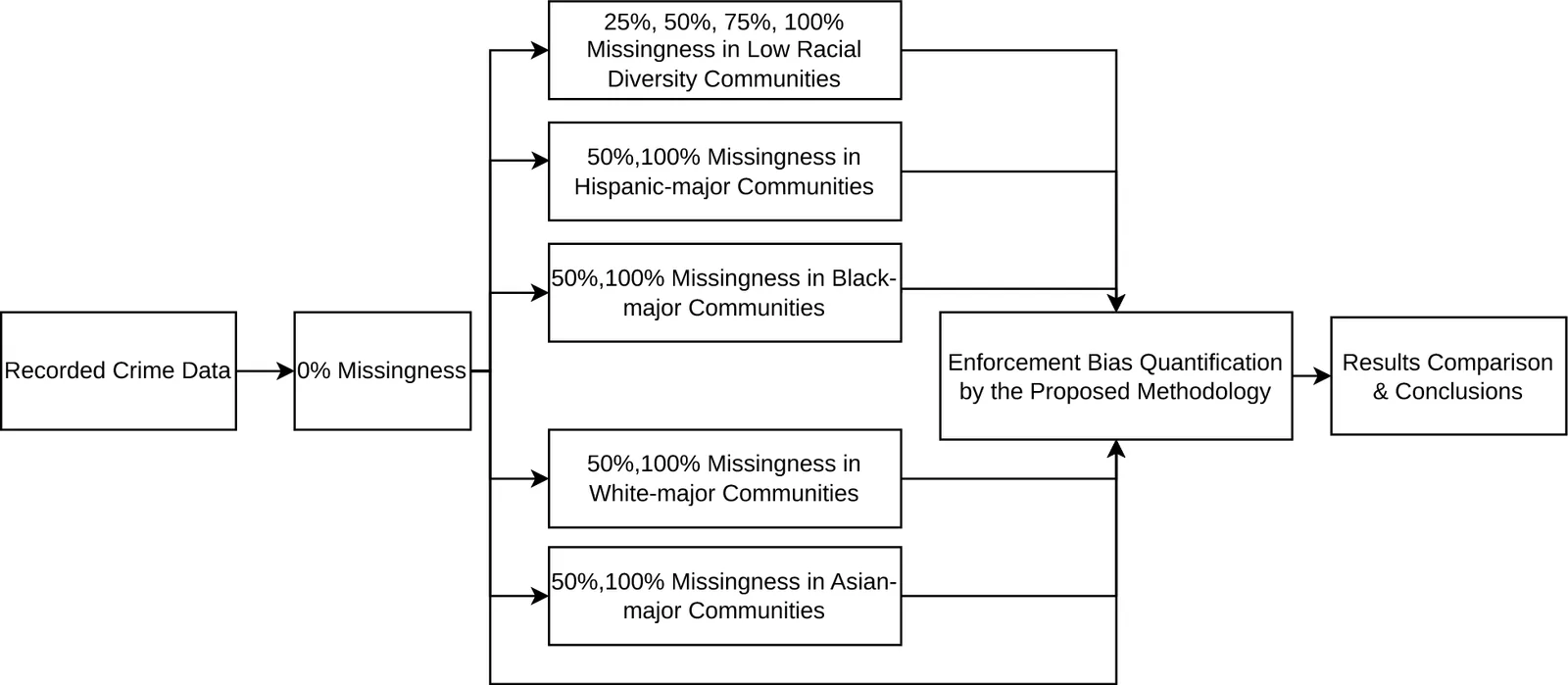
The steps of all the experiments are designed as shown. There are six scenarios in total: the baseline (0% missingness), low racial diversity communities (25%, 50%, 75%, 100%), Hispanic-major communities (50%, 100%), Black-major communities (50%, 100%), White-major communities (50%, 100%), and Asian-major communities (50%, 100%). The five missingness scenarios correspond to 12 reduction settings

The experiments are structured in three steps to address the research questions posed in Section “Introduction”. The flow diagram of the experiments is displayed as Fig. 1.

*Step 1: Baseline prediction (0% missingness).* We train HAGEN on the original Chicago crime data (2022–2024), using January 2022–May 2024 for training and June–August 2024 for validation. The four months of September, October, November, and December 2024 serve as separate test periods. This baseline establishes HAGEN’s performance under complete data.^5^

*Step 2: Controlled data reduction.* To address $RQ_\textrm{2}$, we systematically remove crime records from two types of communities: (i) *low racial diversity communities*, where we apply reductions of 25%, 50%, 75%, and 100%; then (ii) we consider four additional scenarios (one per ethnicity) involving *single-ethnicity-major communities* (Hispanic-, Black-, White-, and Asian-major), where we apply reductions of 50% and 100%. All reductions are performed by random sampling. Specifically, for each scenario, the specified fraction of crime events is removed by uniform random sampling within the targeted subset of communities. This design is a controlled stress test and not a simulation of a real missingness mechanism. The non-random element is *where* records are removed, as missingness is targeted at communities selected by their demographic composition. Within a targeted community, removal is uniform, so that the effect of the *amount* of missing data is separated from the effect of its *location*. Real missingness is not at random and also depends on crime type, victim–offender relationship, and willingness to report (Buil-Gil et al., 2022; Fogliato et al., 2024; Xie et al., 2023), and the direction of the induced bias depends on the missingness pattern (Stockton et al., 2024). The scenarios must therefore be read as directional evidence on how PP outputs react to incompleteness, and not as estimates of the disparities produced by real-world missingness.

*Step 3: Enforcement bias quantification.* We apply the methodology from Section “Methodology” to each reduced dataset, computing over- and under-prediction proportions for every ethnic group ($RQ_\textrm{1}$). Comparing these proportions across reduction scenarios then yields insights into how different types of data incompleteness affect each ethnicity ($RQ_\textrm{2}$).

All experiments were implemented in Python 3.7 and run on a machine with a 13th-generation Intel Core i7-1355U processor (1.70 GHz, 64-bit). This yields six scenarios in total: the original (complete-data) baseline plus five missingness scenarios, i.e., the low racial diversity communities scenario and one scenario for each of the four single-ethnicity-major community types listed in Step 2. The five missingness scenarios comprise 12 reduction settings: four for low racial diversity communities (25%, 50%, 75%, and 100%) and two for each single-ethnicity-major community type (50% and 100%).

### Output and results analysis

**Fig. 2:**
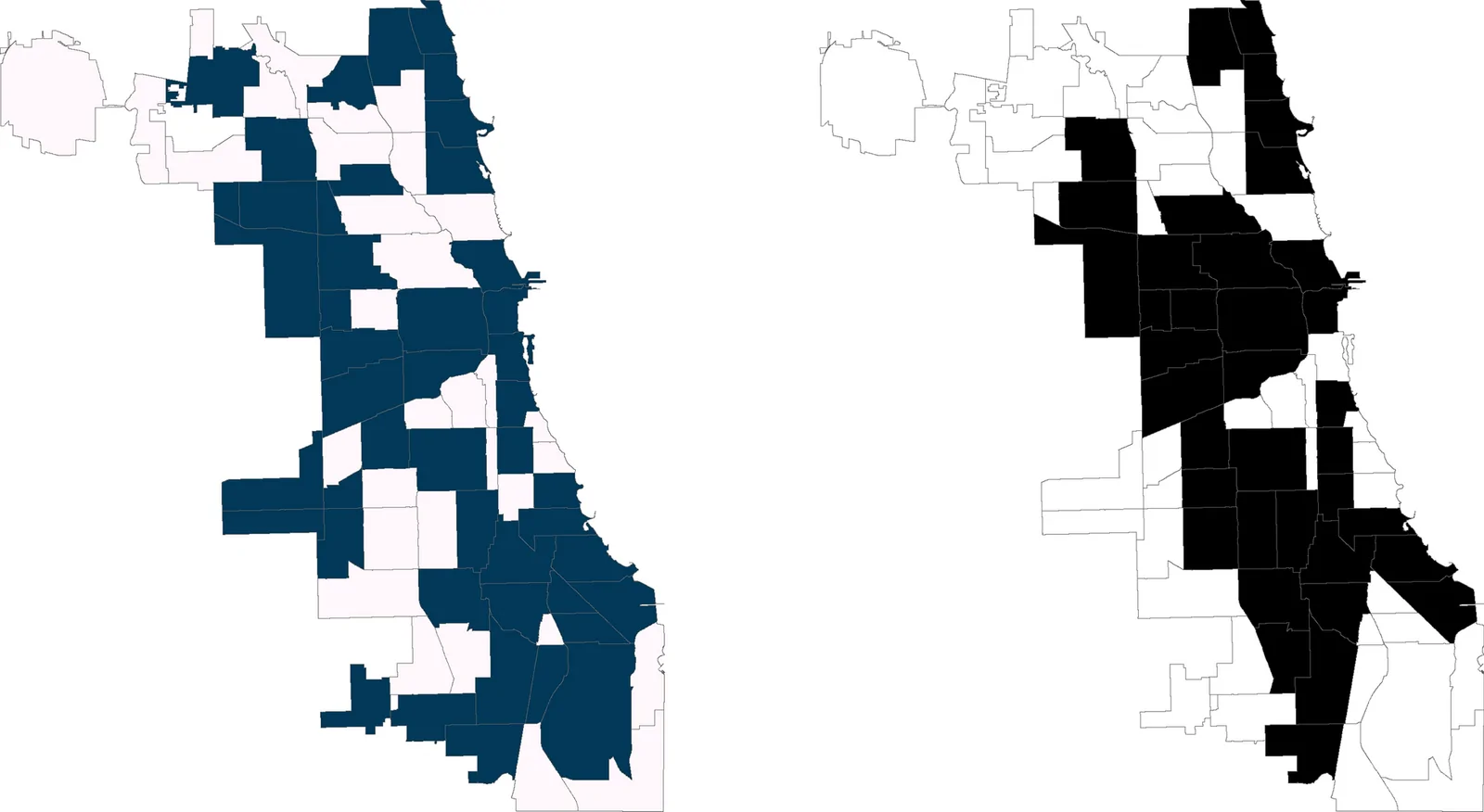
Actual narcotics (left) and predicted narcotics (right) on 1st September 2024 in Chicago. The darker colour in some areas represents crimes happen in these areas, while crimes do not happen in other areas

We apply HAGEN to the Chicago data under each scenario from Step 2, producing binary crime predictions for September–December 2024. Figure 2 shows example crime maps for narcotics on 1 September 2024: darker areas indicate where crimes occur (actual or predicted). Differences between actual and predicted outcomes are treated as prediction errors and fed into the enforcement-bias quantification method from Section “Methodology”.

The full experimental outputs are reported in Appendix C, and Appendix B presents the detailed analysis per crime type. Compared to the baseline (0% reduction), Figs. 3, 4, 5, 6, 7, 8, 9 and 10 visualise the changes in the FP and FN proportions of each ethnicity under the 12 reduction settings.

**Fig. 3:**
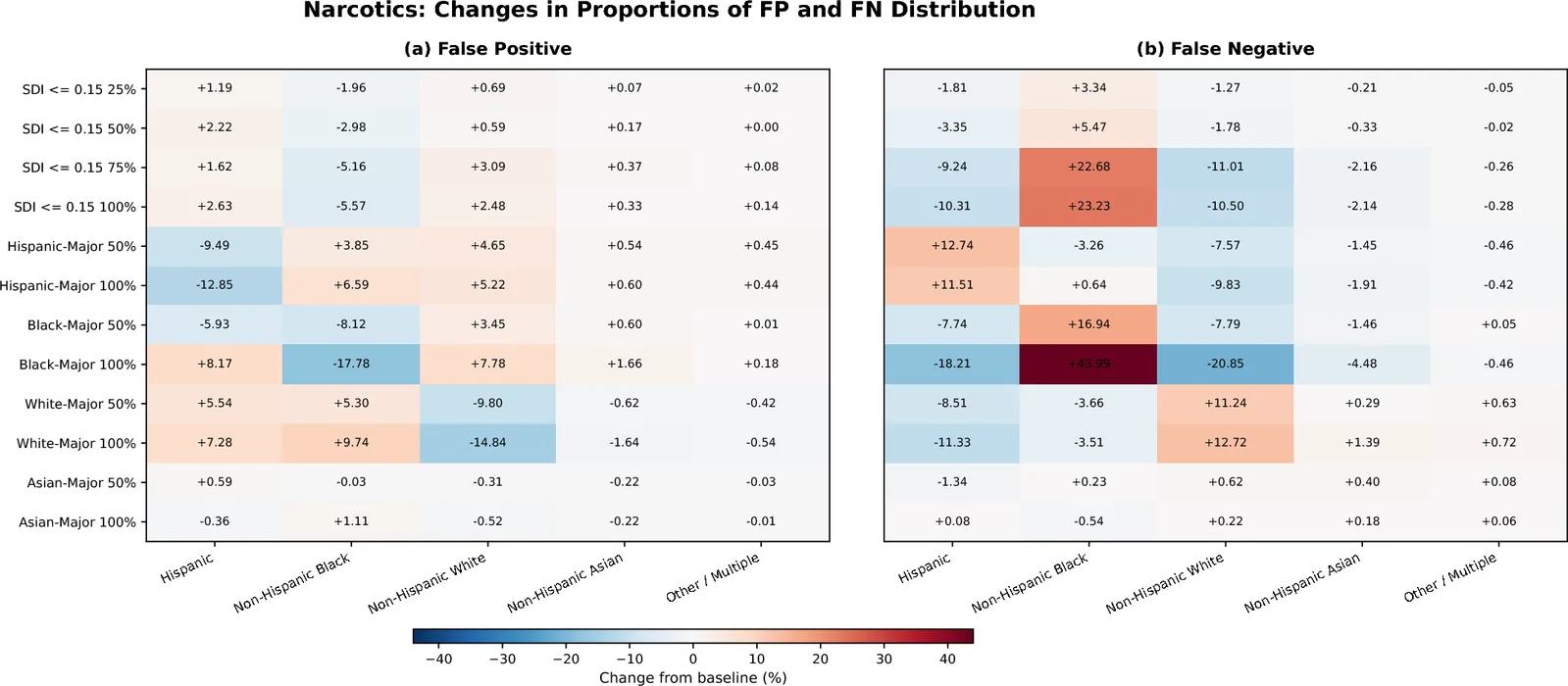
Narcotics: changes of FP/FN proportions for each ethnicity in September­–December 2024 under the 12 reduction settings. Dark blue indicates a large decrease with respect to the baseline, dark red a large increase, and pale cells small changes

**Fig. 4:**
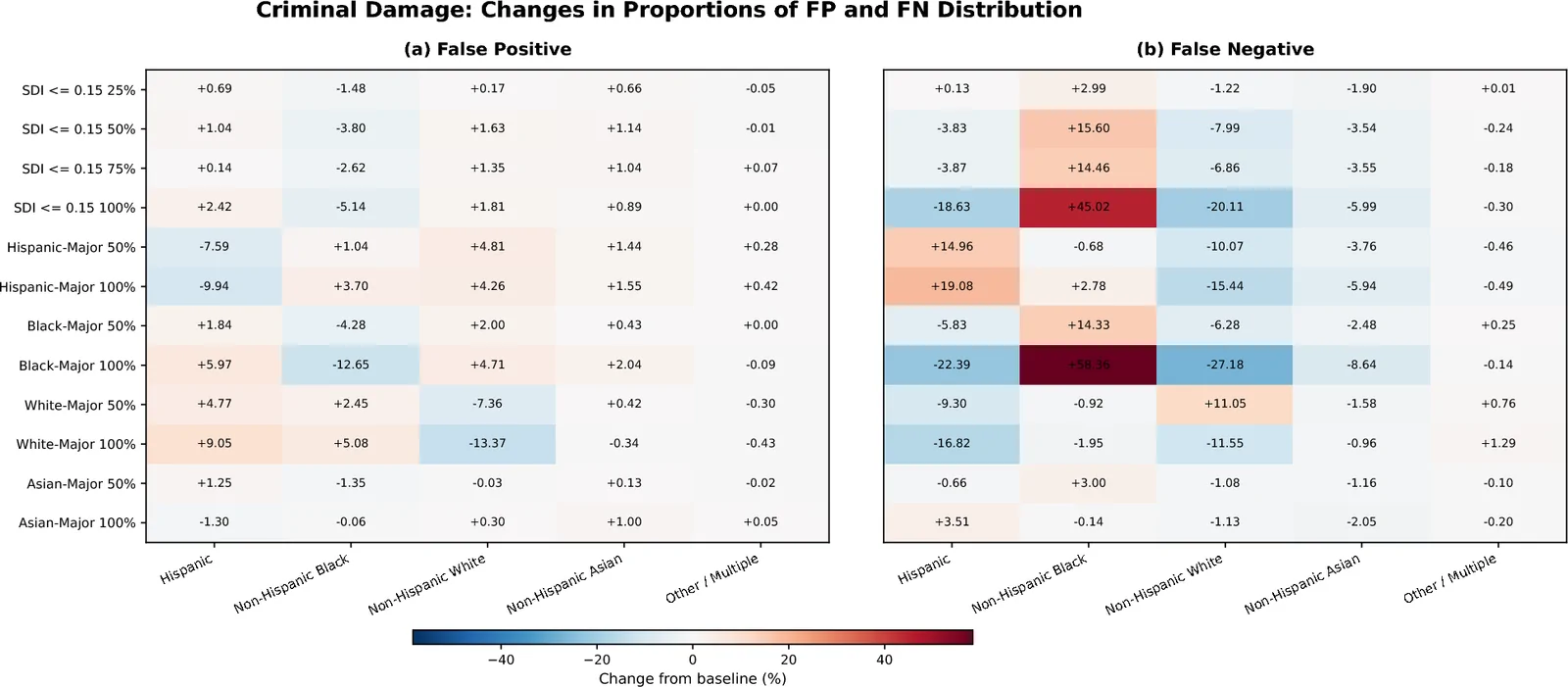
Criminal damage: changes of FP/FN proportions for each ethnicity in September–December 2024 under the 12 reduction settings. Dark blue indicates a large decrease with respect to the baseline, dark red a large increase, and pale cells small changes

**Fig. 5:**
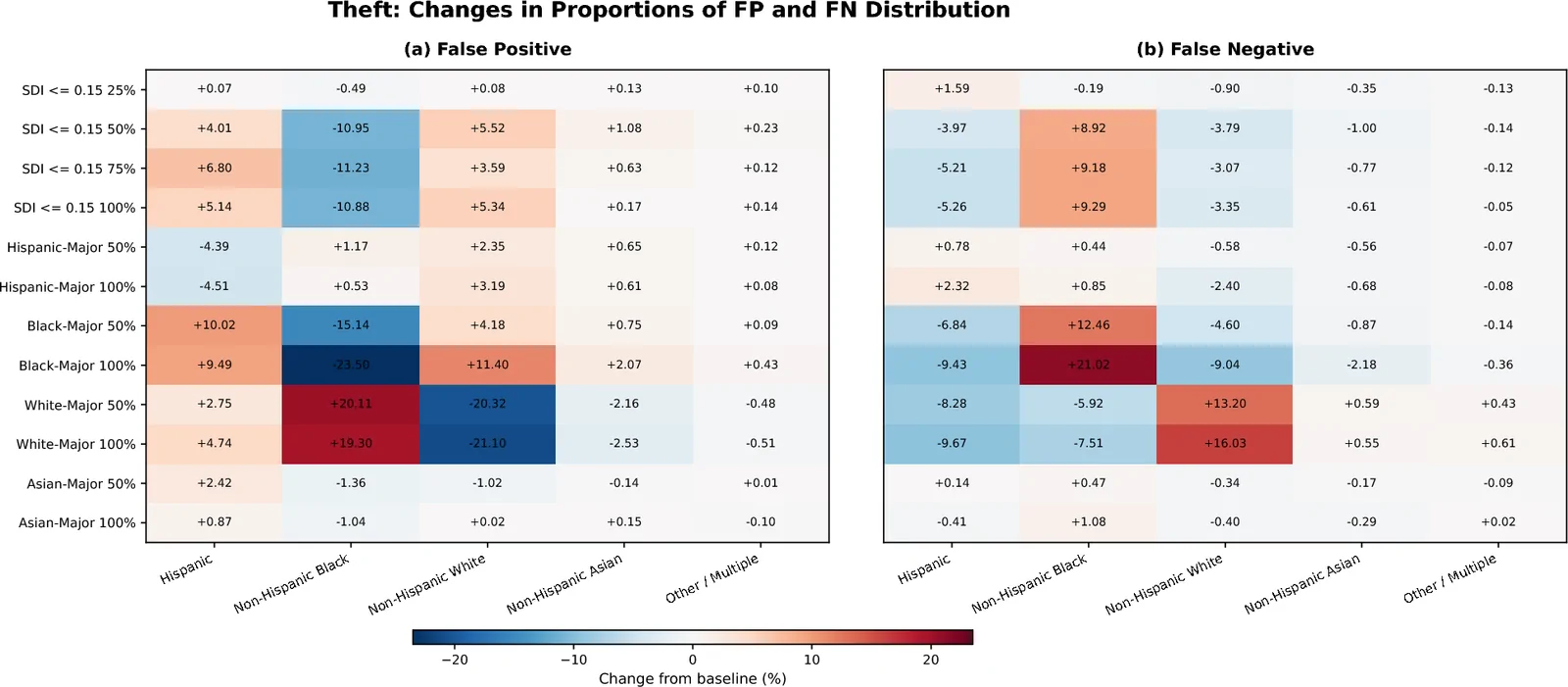
Theft: changes of FP/FN proportions for each ethnicity in September–December 2024 under the 12 reduction settings. Dark blue indicates a large decrease with respect to the baseline, dark red a large increase, and pale cells small changes

**Fig. 6:**
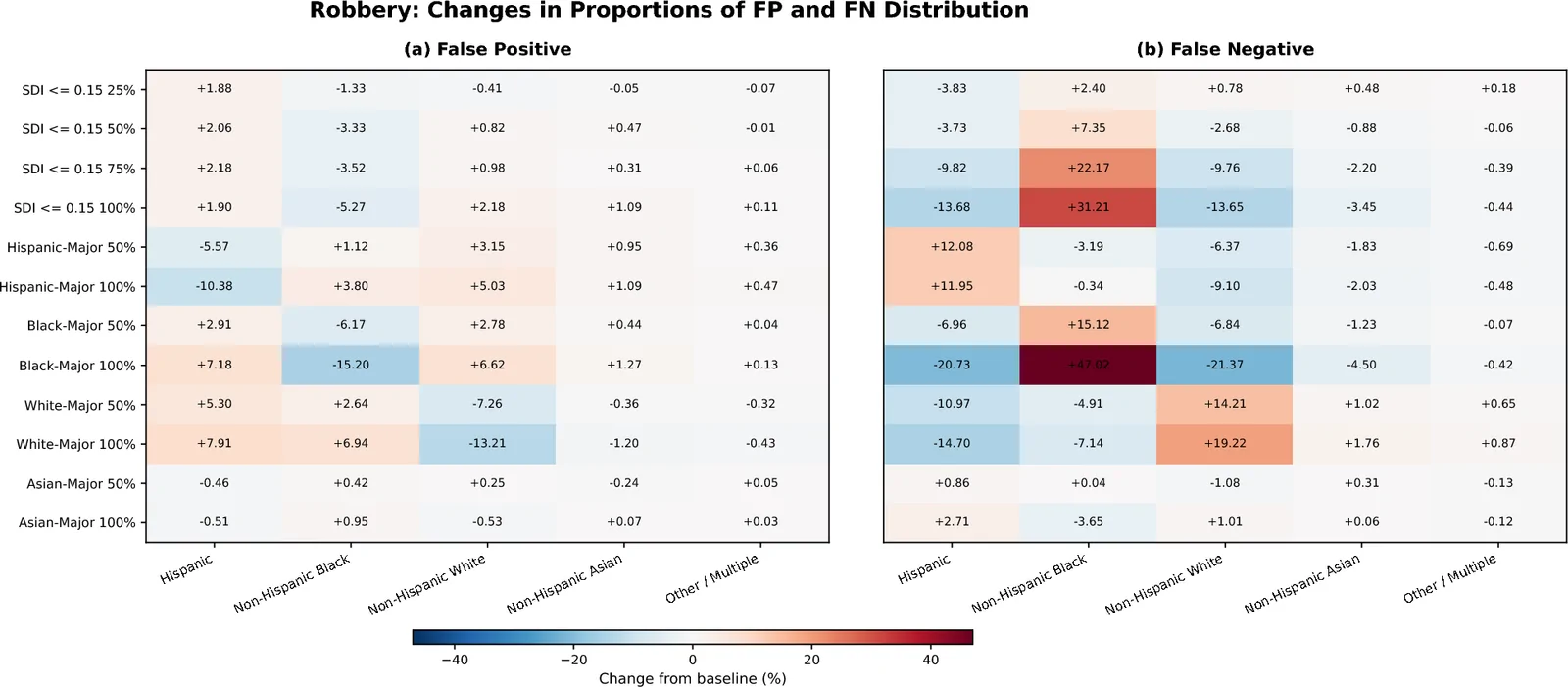
Robbery: changes of FP/FN proportions for each ethnicity in September–December 2024 under the 12 reduction settings. Dark blue indicates a large decrease with respect to the baseline, dark red a large increase, and pale cells small changes

**Fig. 7:**
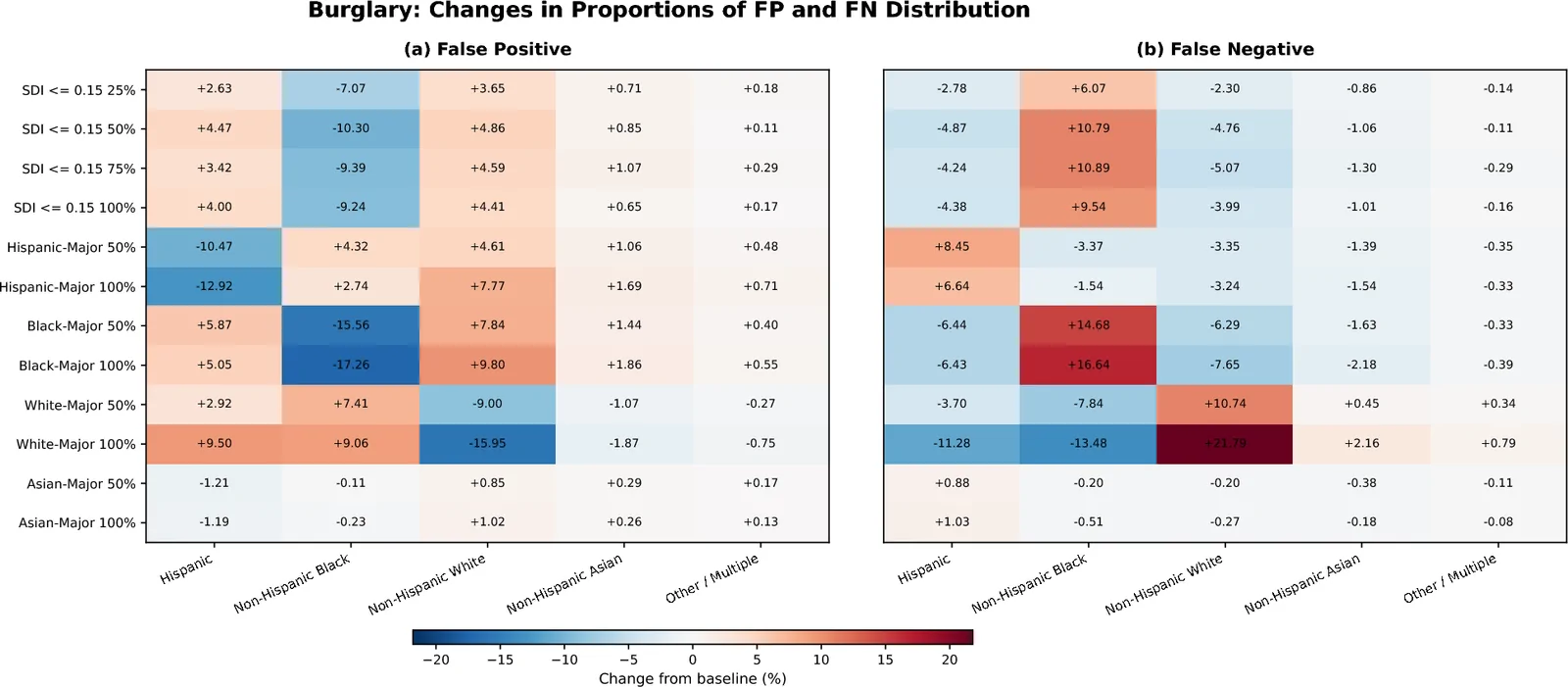
Burglary: changes of FP/FN proportions for each ethnicity in September–December 2024 under the 12 reduction settings. Dark blue indicates a large decrease with respect to the baseline, dark red a large increase, and pale cells small changes

**Fig. 8:**
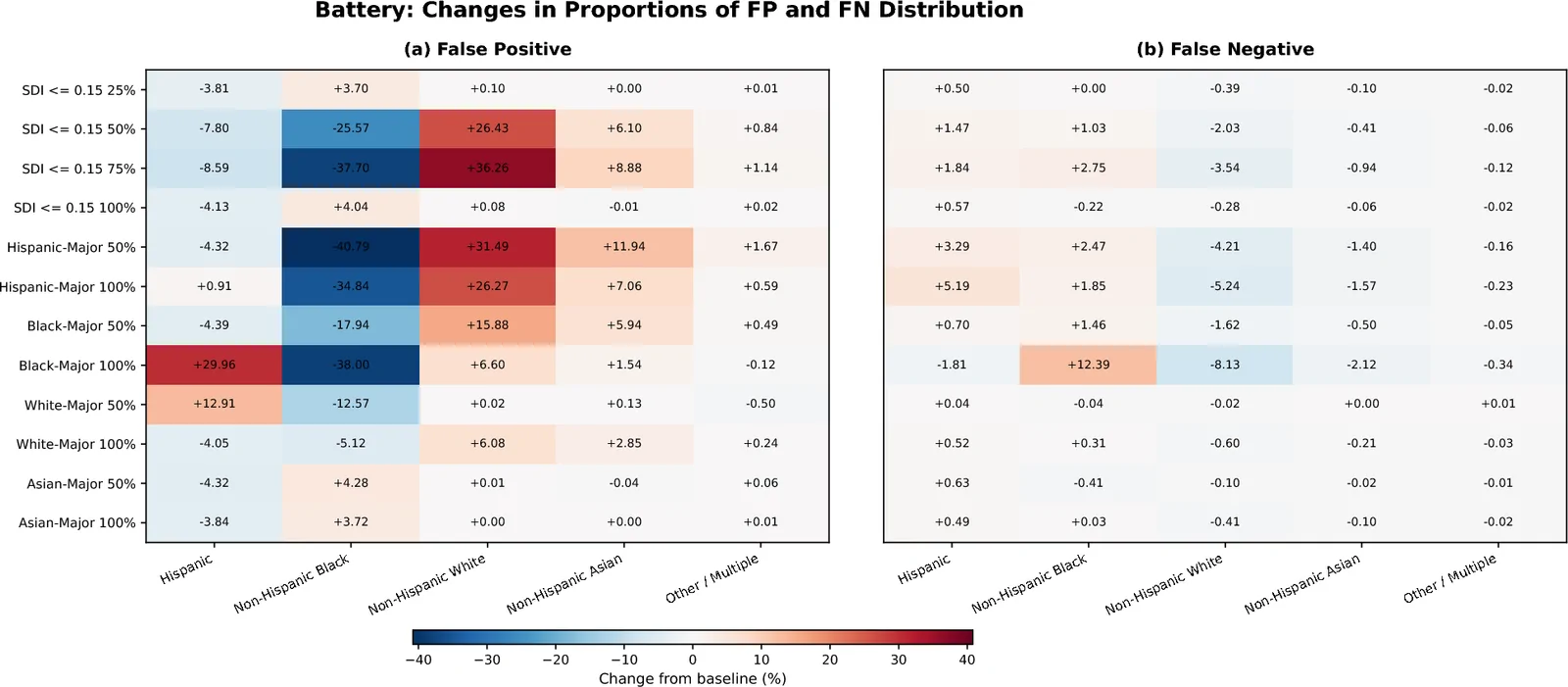
Battery: changes of FP/FN proportions for each ethnicity in September–December 2024 under the 12 reduction settings. Dark blue indicates a large decrease with respect to the baseline, dark red a large increase, and pale cells small changes

**Fig. 9:**
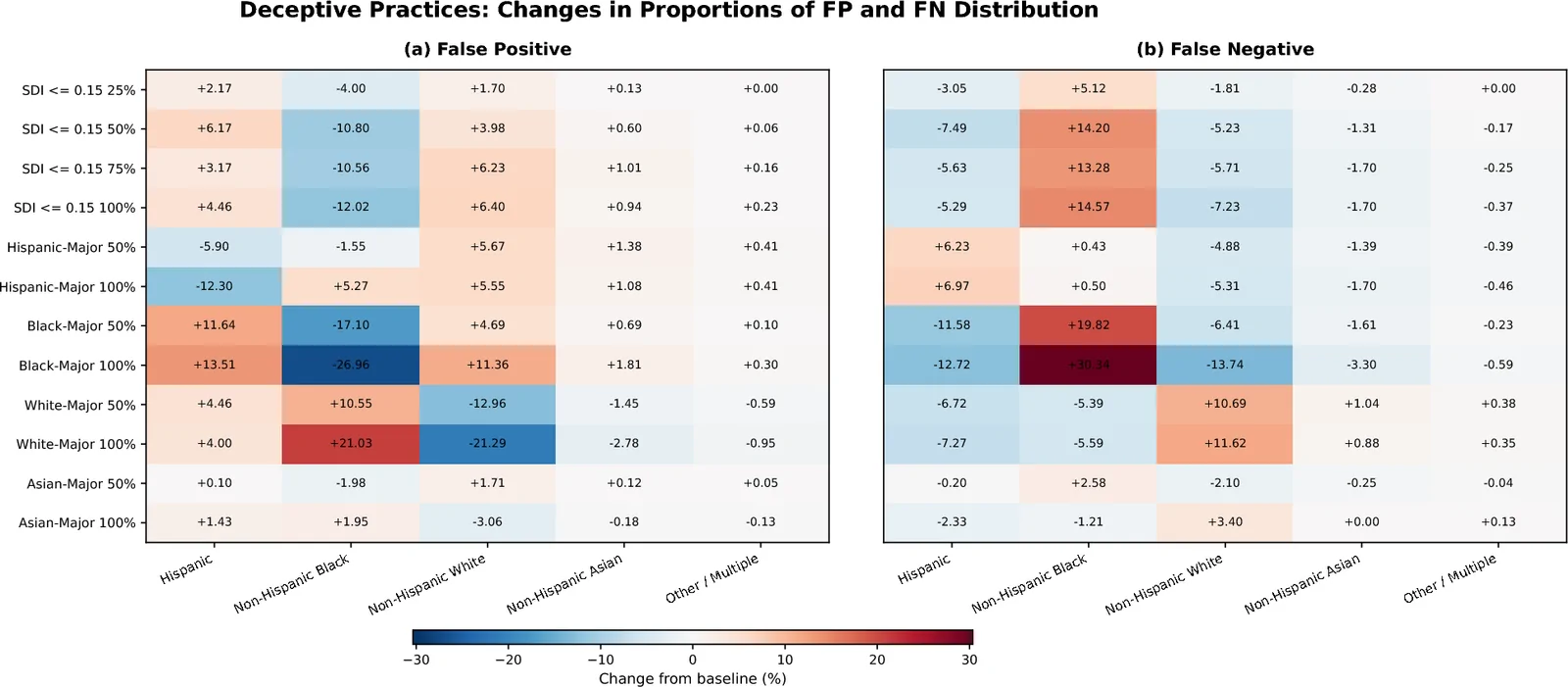
Deceptive practices: changes of FP/FN proportions for each ethnicity in September–December 2024 under the 12 reduction settings. Dark blue indicates a large decrease with respect to the baseline, dark red a large increase, and pale cells small changes

**Fig. 10:**
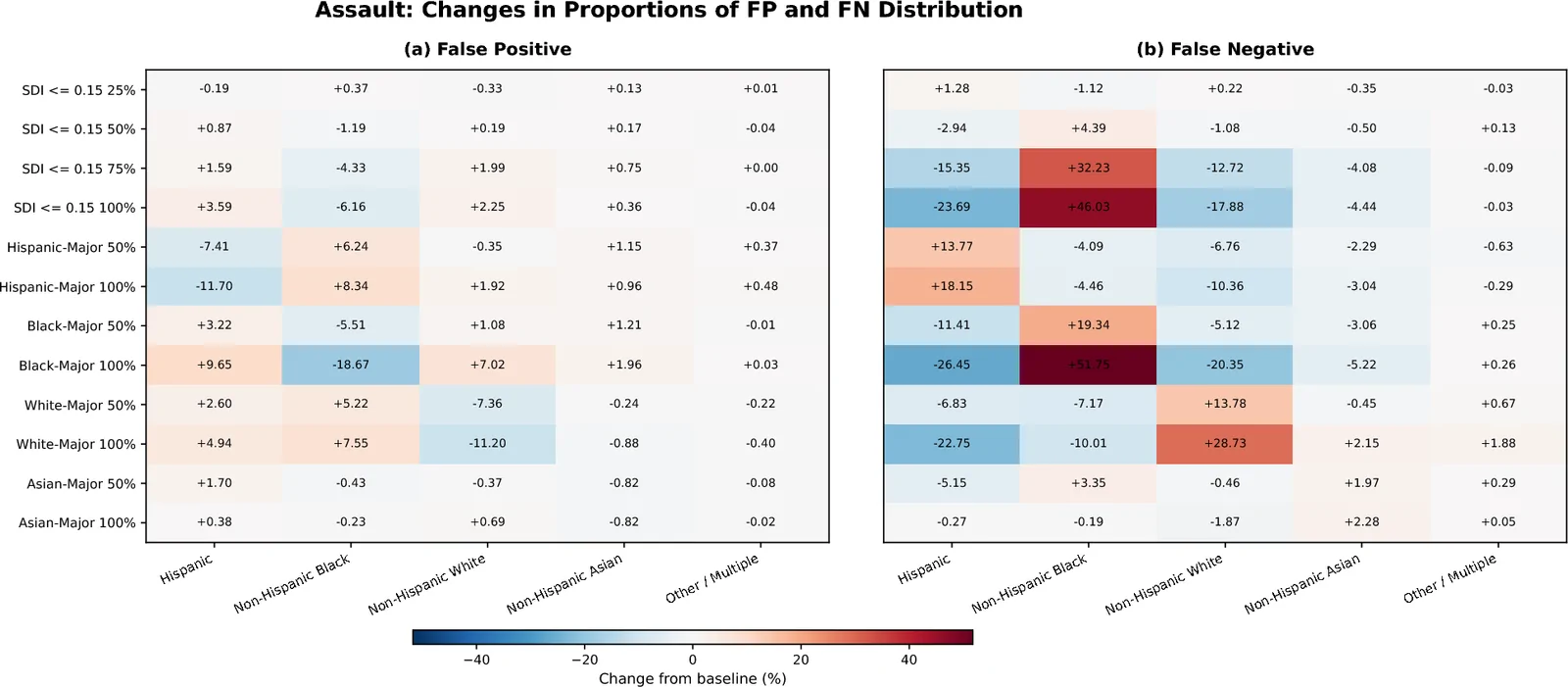
Assault: changes of FP/FN proportions for each ethnicity in September–December 2024 under the 12 reduction settings. Dark blue indicates a large decrease with respect to the baseline, dark red a large increase, and pale cells small changes

By analysing the figures, four cross-cutting findings emerge from the full set of results.

1. *The low racial diversity communities scenario amplifies Black enforcement bias.* As missingness increases in low-SDI areas, predicted over-policing for Black residents falls while predicted under-policing rises; the opposite trend holds for other ethnicities. Across all eight crime types, data incompleteness in these communities produces the most negative policing outcomes for Black residents.
2. *The single-ethnicity-majority communities scenarios shift bias toward the majority group.* Increased missingness in a *e*-majority community generally reduces predicted over-policing and increases predicted under-policing for ethnicity *e*, while reversing these effects for others. The one consistent exception is Asian-major communities, where changes across all groups remain small.
3. *Black communities face the largest shifts overall.* Across narcotics, criminal damage, theft, robbery, deceptive practices, and assault, enforcement bias for Black residents changes the most when data are removed from Black-major communities. By contrast, Non-Hispanic Other or Multiple Races consistently shows the smallest bias changes, typically below 1.0%.
4. *Crime type matters.* Battery is the clearest outlier: data reduction in Hispanic-major and White-major communities produces large enforcement-bias changes for Black and Hispanic residents, i.e., groups that do not dominate those communities. This underscores that missing data in one demographic context can amplify disparities for other groups.

## Discussion and conclusions

This paper studies how incomplete crime records affect the fairness of spatio-temporal PP. We reviewed PP models for spatio-temporal crime forecasting and prior work on PP fairness. The review shows that little work examines how missing crime data changes prediction outcomes across ethnic groups. We therefore posed two research questions.

### Discussion

In Section “Case study”, we ran experiments under the complete-data baseline and five missingness scenarios, i.e., 12 reduction settings. Table 2 (Appendix A) shows that HAGEN achieves good overall accuracy, but prediction errors remain. These errors may arise from the model, from the underlying data, or from both; our methodology captures their combined effect on predicted policing exposure without attributing them to a single source. To study how these errors translate into group-level disparities under different missingness conditions, we proposed a method to quantify enforcement bias by ethnicity. This addresses $RQ_\textrm{1}$. We then compare enforcement bias across crime types and scenarios to address $RQ_\textrm{2}$.

Across scenarios, we find that increasing data incompleteness in single-ethnicity-major communities can increase enforcement bias for the majority group (with Asian residents as an exception in our data). The largest changes appear for Black communities. These shifts occur in both directions: predicted over-policing and predicted under-policing. We also find that the effect depends on the crime type. For example, removing battery records in Hispanic-major communities produces large changes in predicted over-policing for both Black and White communities. More generally, missing data in one demographic context can amplify inferred impacts for other groups, depending on the crime type. On the other hand, the reasons why enforcement bias shifts most markedly for Black residents under missingness in Black-major communities are an interesting question that goes beyond the scope of this paper, which focuses on quantifying rather than explaining these disparities. We flag this as a priority direction for future research. We also note that empirical research has found race to be unrelated to violent crime once community-level socio-economic factors are controlled (Henderson et al., 2024); our use of neighbourhood ethnicity composition as a structural proxy variable does not imply a causal relationship between ethnicity and crime.

These results show that incomplete records can propagate into PP outputs in ways that create or increase ethnic disparities. They are model-based proxies of policing exposure, driven by prediction errors under controlled incompleteness. They are not measurements of police behaviour. Their value is therefore diagnostic: they show when and where the outputs of a PP system become sensitive to the quality of its input data.

### Conclusions

This work makes three contributions. First, we provide a neighbourhood-level method to quantify enforcement bias by ethnicity from spatio-temporal prediction errors, linking false positives and false negatives to potential over- and under-policing. Second, we design controlled data-reduction experiments that vary missingness across demographic contexts, including low-diversity areas and single-ethnicity-major communities. Third, using Chicago data (2022–2024) and HAGEN, we show that data incompleteness can systematically shift over-/under-prediction disparities across ethnic groups.

This paper is an auditing study on the impact of missing crime data on different ethnicities. Its main conclusions are the following:

- Incomplete records can propagate into PP outputs in ways that create or increase ethnic disparities. The direction of the shift depends on where missingness concentrates.
- The largest shifts are observed for Black residents, both when records are missing in low racial diversity communities and when they are missing in Black-major communities.
- Before any operational use, PP systems should include systematic audits of missingness and of its demographic distribution.
- PP systems should also report over- and under-prediction disparities by ethnic group, as these are the quantities that translate into potential policing exposure.
- When data quality is uncertain, predictions should be treated as sensitive to missingness, and the data collection and recording process should be documented.

### Limitations and future work

This study has several limitations. First, in the case study we treat recorded crimes as a proxy for crimes that occurred. This is not generally true, but it lets us isolate how PP outputs change when records are missing. Second, our enforcement-bias measures are derived from prediction errors. They represent potential misallocation of policing attention under the assumption that predictive policing outputs are used to inform enforcement actions, not observed enforcement actions. Third, our controlled missingness does not model real missingness mechanisms, such as non-random under-reporting or differential recording. Records are removed uniformly at random within the targeted communities, so the only non-random element of the design is the choice of the communities themselves. Real missingness also varies with crime type, victim–offender relationship, and willingness to report, and the direction of the induced bias depends on that pattern (Stockton et al., 2024). Our scenarios are therefore a controlled stress test and not a representation of real-world missingness. In addition, the Chicago records used as a baseline are themselves incomplete, so the reported changes are shifts with respect to an already incomplete starting point and are likely to understate the total distortion affecting a deployed system. Finally, we study one city (Chicago), one period (2022–2024), a fixed set of crime types, and one model (HAGEN), so external validity is limited. In particular, as with any chronological evaluation, the train/validation/test split spans seasons. Results may therefore differ if the model were tested on other months, and this should be taken into account when generalising findings beyond the autumn and winter period.

Future work could model non-random missingness mechanisms directly. It could also validate results with independent measures of crime and reporting, such as victimisation surveys or calls-for-service. Another direction is to test whether the same patterns hold in other cities, time periods, and PP models. Future work could also use additional fairness metrics and uncertainty estimates to identify when PP outputs become unreliable under incomplete data. Finally, as the results of this paper show, differences in enforcement bias and data incompleteness across scenarios and ethnic groups represent a noteworthy direction for PP research and may help inform policing strategies.

We hope this work encourages further scrutiny of how data quality interacts with fairness in PP and, ultimately, how that interaction affects the communities these systems are meant to serve.

## Data Availability

Crime incident data used in this study arein the public. Chicago crime data is obtained from https://data.cityofchicago.org/.The demographic data of Chicago used for this study is available athttps://www.actforchildren.org/wp-content/uploads/2024/03/Census-Data-by-Chicago-Community-Area-2022.pdf.The boundaries information of Chicago is available athttps://data.cityofchicago.org/Facilities-Geographic-Boundaries/Boundaries-Community-Areas-Map/cauq-8yn6.

## Performance of HAGEN

In Section “Case study”, this paper has explored the performance of all data scenarios by HAGEN. Table 2 presents all the Micro-F1 and Macro-F1 values of each month.

**Table 2:** Model performance under different reduction scenarios

Scenario	Month	Micro-F1	Macro-F1
0% Reduction	9	0.71274	0.61821
	10	0.70767	0.62076
	11	0.60807	0.58170
	12	0.61976	0.59349
25% Reduction in low racial diversity communities	9	0.70970	0.55824
	10	0.70892	0.55215
	11	0.67649	0.52062
	12	0.68306	0.53821
50% Reduction in low racial diversity communities	9	0.70027	0.64228
	10	0.70130	0.64147
	11	0.67206	0.61107
	12	0.67998	0.61715
75% Reduction in low racial diversity communities	9	0.68165	0.64064
	10	0.68251	0.63582
	11	0.65789	0.60497
	12	0.66797	0.59556
100% Reduction in low racial diversity communities	9	0.66917	0.59974
	10	0.66278	0.59753
	11	0.63962	0.5636
	12	0.63963	0.57706
50% Reduction in Hispanic-major communities	9	0.68959	0.62741
	10	0.6913	0.62955
	11	0.66443	0.59573
	12	0.67215	0.60701
100% Reduction in Hispanic-major communities	9	0.64806	0.57299
	10	0.66006	0.58076
	11	0.61443	0.54484
	12	0.62746	0.56001
50% Reduction in black-major communities	9	0.67721	0.60639
	10	0.68252	0.60454
	11	0.6548	0.5771
	12	0.66427	0.5923
100% Reduction in black-major communities	9	0.52788	0.46068
	10	0.51823	0.46354
	11	0.51111	0.43709
	12	0.52356	0.44897
50% Reduction in white-major communities	9	0.68878	0.61821
	10	0.69486	0.62076
	11	0.66991	0.5817
	12	0.66658	0.59349
100% Reduction in white-major communities	9	0.61497	0.55824
	10	0.61532	0.55215
	11	0.58963	0.52062
	12	0.60529	0.53821
50% Reduction in Asian-major communities	9	0.71237	0.64228
	10	0.71073	0.64147
	11	0.68079	0.61107
	12	0.68343	0.61715
100% Reduction in Asian-major communities	9	0.71095	0.64064
	10	0.71302	0.63582
	11	0.67994	0.60497
	12	0.68224	0.61778

## Detailed output of experiments

Throughout this appendix, references to over-policing and under-policing denote predicted quantities derived from model errors, as defined in Section “Methodology”, and should be interpreted as proxies for potential policing exposure rather than observed enforcement actions.

### Narcotics

Results are shown in Table 3 and 4. Narcotics serve as the reference crime type; patterns for most other types broadly follow those described here.

- *Over-policing.* In the low racial diversity communities scenario, greater missingness increases over-policing for all ethnicities *except* Black residents: Black residents see a 5.57% *decrease* as incompleteness rises from 0 to 100%. In the single-ethnicity-major communities scenarios over-policing for the majority ethnicity falls while it rises for others; for example, Hispanic over-policing drops steadily from 29.94 to 17.09% as data reduction in Hispanic-major communities goes from 0 to 100%.
- *Under-policing.* In the low racial diversity communities scenario, greater missingness reduces under-policing for all ethnicities except Black residents, whose under-policing surges from 16.67 to 39.9%. In the single-ethnicity-major communities scenarios, under-policing for the majority ethnicity increases with missingness. The exception is Asian-major communities, where changes across all five groups remain below 1.42%.

### Criminal damage, theft, robbery, burglary, and assault

Results for these five crime types are shown in Tables 5, 6, 7, 8, 9, 10, 11, 12, 17 and 18. The patterns closely mirror those for narcotics; we note the key deviations below.

- *Criminal damage.* The sharpest under-policing increase occurs when data in Black-major communities are reduced: under-policing for Black residents rises by 58.36% from 0% to 100% reduction. In the low racial diversity communities scenario, under-policing for Black residents rises from 11.26% to 56.28%. Under 100% reduction in Asian-major communities, over-policing for Asian residents is approximately 1% *higher* than under complete data, i.e., the opposite of the usual ethnicity-major scenario pattern.
- *Theft.* Over-policing for White and Black residents each increase by more than 20% when missingness reaches 100% in the corresponding majority communities. In Hispanic-major and Asian-major community scenarios, under-policing changes remain small (*≤ *2.4% and *≤ *1.08%, respectively).
- *Robbery.* The largest over-policing shift occurs in Black-major communities (Black residents: −15.2%). Under-policing in the low racial diversity scenario and in the Black-major communities scenario increases by 31.21% and 47.02%, respectively. Over-policing changes in the Asian-major communities scenario are below 1.0% for every ethnicity.
- *Burglary.* The largest over-policing shift again occurs in the Black-major communities scenario (Black residents: −17.26%). Under-policing for White residents rises by 21.79% in the White-major communities scenario; all other scenarios produce changes below 20%.
- *Assault.* The largest changes occur in the Black-major communities scenario: over-policing for Black residents falls by 18.67%, while under-policing rises by 51.75%.

### Battery

Battery departs from the general pattern and warrants separate discussion (Tables 13 and 14).

- *Over-policing.* In the low racial diversity communities scenarios, results oscillate rather than trend monotonically. Over-policing for Black residents falls sharply from 72.65 to 34.95% as reductions go from 0 to 75%, but then rebounds to 76.69% at 100% reduction. White residents follow the inverse path: rising from 5.28% to 41.54% at 75% before returning to 5.36% at 100%. In the Hispanic-major communities scenario, Black residents’ over-policing decreases the most (−40.79% at 50% reduction). In the Black-major communities scenario, Black residents’ over-policing falls steadily by 38%, while other ethnicities reach extreme values at 50% reduction. In the White-major communities scenario, Black and Hispanic residents are most affected (changes of 16.96% and 12.57%), while White residents change by only 6.08%. The Asian-major scenarios produce no notable changes (*≤ *4.32%).
- *Under-policing.* Changes in low racial diversity, White-major, and Asian-major communities scenarios are negligible (*≤ *3.54%). In Black-major and Hispanic-major communities scenarios, under-policing trends resemble those for narcotics.

### Deceptive practice

Results are shown in Tables 15 and 16. Patterns under the low racial diversity, Hispanic-, White-, and Black-major scenarios broadly follow those for narcotics, with two notable findings.

- Increasing missingness in Black-major communities reduces over-policing for Black residents by 26.96% and raises under-policing by 30.34%, i.e., the largest over-policing and under-policing change across all scenarios.
- In the Asian-major communities scenario, data reduction has little effect on Asian residents’ under-policing, but the step from 50% to 100% reduction produces a 5.5% increase in White residents’ over-policing, i.e., the largest ethnicity-level change under that scenario.

## Results of quantitative bias on each ethnicity of each crime type

The results of quantitative bias on each ethnicity of each crime are shown in this appendix.

**Table 3:** Narcotics: proportion results of false positive on each ethnicity in September–December 2024

Scenario	Hispanic (%)	Black (%)	White (%)	Asian (%)	Other races (%)
0% Reduction	29.94	28.67	31.73	6.41	3.25
25% Reduction in low racial diversity communities	31.13	26.71	32.42	6.48	3.27
50% Reduction in low racial diversity communities	32.16	25.69	32.32	6.58	3.25
75% Reduction in low racial diversity communities	31.56	23.51	34.82	6.78	3.33
100% Reduction in low racial diversity communities	32.57	23.1	34.21	6.74	3.39
50% Reduction in Hispanic-major communities	20.45	32.52	36.38	6.95	3.7
100% Reduction in Hispanic-major communities	17.09	35.26	36.95	7.01	3.69
50% Reduction in black-major communities	24.01	20.55	35.18	7.01	3.26
100% Reduction in black-major communities	38.11	10.89	39.51	8.07	3.43
50% Reduction in white-major communities	35.48	33.97	21.93	5.79	2.83
100% Reduction in white-major communities	37.22	38.41	16.89	4.77	2.71
50% Reduction in Asian-major communities	30.53	28.64	31.42	6.19	3.22
100% Reduction in Asian-major communities	29.58	29.78	31.21	6.19	3.24

**Table 4:** Narcotics: proportion results of false negative on each ethnicity in September–December 2024

Scenario	Hispanic (%)	Black (%)	White (%)	Asian (%)	Other races (%)
0% Reduction	36.19	16.67	36.4	7.86	2.88
25% Reduction in low racial diversity communities	34.38	20.01	35.13	7.65	2.83
50% Reduction in low racial diversity communities	32.84	22.14	34.62	7.53	2.86
75% Reduction in low racial diversity communities	26.95	39.35	25.39	5.7	2.62
100% Reduction in low racial diversity communities	25.88	39.9	25.9	5.72	2.6
50% Reduction in Hispanic-major communities	48.93	13.41	28.83	6.41	2.42
100% Reduction in Hispanic-major communities	47.7	17.31	26.57	5.95	2.46
50% Reduction in black-major communities	28.45	33.61	28.61	6.4	2.93
100% Reduction in black-major communities	17.98	60.66	15.55	3.38	2.42
50% Reduction in white-major communities	27.68	13.01	47.64	8.15	3.51
100% Reduction in white-major communities	24.86	13.16	49.12	9.25	3.6
50% Reduction in Asian-major communities	34.85	16.9	37.02	8.26	2.96
100% Reduction in Asian-major communities	36.27	16.13	36.62	8.04	2.94

**Table 5:** Criminal damage: results of false positive on each ethnicity in September–December 2024

Scenario	Hispanic (%)	Black (%)	White (%)	Asian (%)	Other races (%)
0% Reduction	32.02	20.65	36.88	7.13	3.33
25% Reduction in low racial diversity communities	32.71	19.17	37.05	7.79	3.28
50% Reduction in low racial diversity communities	33.06	16.85	38.51	8.27	3.32
75% Reduction in low racial diversity communities	32.16	18.03	38.23	8.17	3.4
100% Reduction in low racial diversity communities	34.44	15.51	38.69	8.02	3.33
50% Reduction in Hispanic-major communities	24.43	21.69	41.69	8.57	3.61
100% Reduction in Hispanic-major communities	22.08	24.35	41.14	8.68	3.75
50% Reduction in black-major communities	33.86	16.37	38.88	7.56	3.33
100% Reduction in black-major communities	37.99	8	41.59	9.17	3.24
50% Reduction in white-major communities	36.79	23.1	29.52	7.55	3.03
100% Reduction in white-major communities	41.07	25.73	23.51	6.79	2.9
50% Reduction in Asian-major communities	33.27	19.3	36.85	7.26	3.31
100% Reduction in Asian-major communities	30.72	20.59	37.18	8.13	3.38

**Table 6:** Criminal damage: proportion results of false negative on each ethnicity in September–December 2024

Scenario	Hispanic (%)	Black (%)	White (%)	Asian (%)	Other races (%)
0% Reduction	36.34	11.26	38.58	11.31	2.51
25% Reduction in low racial diversity communities	36.47	14.25	37.36	9.41	2.52
50% Reduction in low racial diversity communities	32.51	26.86	30.59	7.77	2.27
75% Reduction in low racial diversity communities	32.47	25.72	31.72	7.76	2.33
100% Reduction in low racial diversity communities	17.71	56.28	18.47	5.32	2.21
50% Reduction in Hispanic-major communities	51.30	10.58	28.51	7.55	2.05
100% Reduction in Hispanic-major communities	55.42	14.04	23.14	5.37	2.02
50% Reduction in black-major communities	30.51	25.59	32.30	8.83	2.76
100% Reduction in black-major communities	13.95	69.62	11.40	2.67	2.37
50% Reduction in white-major communities	27.04	10.34	49.63	9.73	3.27
100% Reduction in white-major communities	19.52	9.31	27.03	10.35	3.80
50% Reduction in Asian-major communities	35.68	14.26	37.50	10.15	2.41
100% Reduction in Asian-major communities	39.85	11.12	37.45	9.26	2.31

**Table 7:** Theft: results of false positive on each ethnicity in September–December 2024

Scenario	Hispanic (%)	Black (%)	White (%)	Asian (%)	Other races (%)
0% Reduction	16.45	37.33	35.87	6.93	3.52
25% Reduction in low racial diversity communities	16.52	36.84	35.95	7.06	3.62
50% Reduction in low racial diversity communities	20.46	26.38	41.39	8.01	3.75
75% Reduction in low racial diversity communities	23.25	26.10	39.46	7.56	3.64
100% Reduction in low racial diversity communities	21.59	26.45	41.21	7.10	3.66
50% Reduction in Hispanic-major communities	12.06	38.50	38.22	7.58	3.64
100% Reduction in Hispanic-major communities	11.94	37.86	39.06	7.54	3.60
50% Reduction in black-major communities	26.47	22.19	40.05	7.68	3.61
100% Reduction in black-major communities	25.94	13.83	47.27	9.00	3.95
50% Reduction in white-major communities	19.20	57.44	15.55	4.77	3.04
100% Reduction in white-major communities	21.19	56.63	14.77	4.40	3.01
50% Reduction in Asian-major communities	18.87	35.97	34.85	6.79	3.53
100% Reduction in Asian-major communities	17.32	36.29	35.89	7.08	3.42

**Table 8:** Theft: proportion results of false negative on each ethnicity in September–December 2024

Scenario	Hispanic (%)	Black (%)	White (%)	Asian (%)	Other races (%)
0% Reduction	37.96	22.41	29.50	7.23	2.89
25% Reduction in low racial diversity communities	39.55	22.22	28.60	6.88	2.76
50% Reduction in low racial diversity communities	33.99	31.33	25.71	6.23	2.75
75% Reduction in low racial diversity communities	32.75	31.59	26.43	6.46	2.77
100% Reduction in low racial diversity communities	32.70	31.70	26.15	6.62	2.84
50% Reduction in Hispanic-major communities	38.74	22.85	28.92	6.67	2.82
100% Reduction in Hispanic-major communities	40.28	23.26	27.10	6.55	2.81
50% Reduction in black-major communities	31.12	34.87	24.90	6.36	2.75
100% Reduction in black-major communities	28.53	43.43	20.46	5.05	2.53
50% Reduction in white-major communities	29.68	16.49	42.70	7.82	3.32
100% Reduction in white-major communities	28.29	14.90	45.53	7.78	3.50
50% Reduction in Asian-major communities	38.10	22.88	29.16	7.06	2.80
100% Reduction in Asian-major communities	37.55	23.49	29.10	6.94	2.91

**Table 9:** Robbery: proportion results of false positive on each ethnicity in September–December 2024

Scenario	Hispanic (%)	Black (%)	White (%)	Asian (%)	Other races (%)
0% Reduction	31.30	26.05	32.75	6.69	3.20
25% Reduction in low racial diversity communities	33.18	24.72	32.34	6.64	3.13
50% Reduction in low racial diversity communities	33.36	22.72	33.57	7.16	3.19
75% Reduction in low racial diversity communities	33.48	22.53	33.73	7.00	3.26
100% Reduction in low racial diversity communities	33.20	20.78	34.93	7.78	3.31
50% Reduction in Hispanic-major communities	25.73	27.17	35.90	7.64	3.56
100% Reduction in Hispanic-major communities	20.92	29.85	37.78	7.78	3.67
50% Reduction in black-major communities	34.21	19.88	35.53	7.13	3.24
100% Reduction in black-major communities	38.48	10.85	39.37	7.96	3.33
50% Reduction in white-major communities	36.60	28.69	25.49	6.33	2.88
100% Reduction in white-major communities	39.21	32.99	19.54	5.49	2.77
50% Reduction in Asian-major communities	30.84	26.47	33.00	6.45	3.25
100% Reduction in Asian-major communities	30.79	27.00	32.22	6.76	3.23

**Table 10:** Robbery: proportion results of false negative on each ethnicity in September–December 2024

Scenario	Hispanic (%)	Black (%)	White (%)	Asian (%)	Other races (%)
0% Reduction	37.96	17.59	34.19	7.46	2.80
25% Reduction in low racial diversity communities	34.13	19.99	34.97	7.94	2.98
50% Reduction in low racial diversity communities	34.23	24.94	31.51	6.58	2.74
75% Reduction in low racial diversity communities	28.14	39.76	24.43	5.26	2.41
100% Reduction in low racial diversity communities	24.28	48.80	20.54	4.01	2.36
50% Reduction in Hispanic-major communities	50.04	14.40	27.82	5.63	2.11
100% Reduction in Hispanic-major communities	49.91	17.25	25.09	5.43	2.32
50% Reduction in black-major communities	31.00	32.71	27.35	6.23	2.73
100% Reduction in black-major communities	17.23	64.61	12.82	2.96	2.38
50% Reduction in white-major communities	26.99	12.68	48.40	8.48	3.45
100% Reduction in white-major communities	23.26	10.45	53.41	9.22	3.67
50% Reduction in Asian-major communities	38.82	17.63	33.11	7.77	2.67
100% Reduction in Asian-major communities	40.67	13.94	35.20	7.52	2.68

**Table 11:** Burglary: proportion results of false positive on each ethnicity in September–December 2024

Scenario	Hispanic (%)	Black (%)	White (%)	Asian (%)	Other races (%)
0% Reduction	28.71	30.30	32.55	5.36	3.09
25% Reduction in low racial diversity communities	31.34	23.23	36.20	6.07	3.27
50% Reduction in low racial diversity communities	33.18	20.00	37.41	6.21	3.20
75% Reduction in low racial diversity communities	32.13	20.91	37.14	6.43	3.38
100% Reduction in low racial diversity communities	32.71	21.06	36.96	6.01	3.26
50% Reduction in Hispanic-major communities	18.24	34.62	37.16	6.42	3.57
100% Reduction in Hispanic-major communities	15.79	33.04	40.32	7.05	3.80
50% Reduction in black-major communities	34.58	14.74	40.39	6.80	3.49
100% Reduction in black-major communities	33.76	13.04	42.35	7.22	3.64
50% Reduction in white-major communities	31.63	37.71	23.55	4.29	2.82
100% Reduction in white-major communities	38.21	39.36	16.60	3.49	2.34
50% Reduction in Asian-major communities	27.50	30.19	33.40	5.65	3.26
100% Reduction in Asian-major communities	27.52	30.07	33.57	5.62	3.22

**Table 12:** Burglary: proportion results of false negative on each ethnicity in September–December 2024

Scenario	Hispanic (%)	Black (%)	White (%)	Asian (%)	Other races (%)
0% Reduction	32.57	30.33	27.49	6.75	2.87
25% Reduction in low racial diversity communities	29.79	36.40	25.19	5.89	2.73
50% Reduction in low racial diversity communities	27.70	41.12	22.73	5.69	2.76
75% Reduction in low racial diversity communities	28.33	41.22	22.42	5.45	2.58
100% Reduction in low racial diversity communities	28.19	39.87	23.50	5.74	2.71
50% Reduction in Hispanic-major communities	41.02	26.96	24.14	5.36	2.52
100% Reduction in Hispanic-major communities	39.21	28.79	24.25	5.21	2.54
50% Reduction in black-major communities	26.13	45.01	21.20	5.12	2.54
100% Reduction in black-major communities	26.14	46.97	19.84	4.57	2.48
50% Reduction in white-major communities	28.87	22.49	38.23	7.20	3.21
100% Reduction in white-major communities	21.29	16.85	49.28	8.91	3.66
50% Reduction in Asian-major communities	33.45	30.13	27.29	6.37	2.76
100% Reduction in Asian-major communities	33.60	29.82	27.22	6.57	2.79

**Table 13:** Battery: proportion results of false positive on each ethnicity in September–December 2024

Scenario	Hispanic (%)	Black (%)	White (%)	Asian (%)	Other races (%)
0% Reduction	19.54	72.65	5.28	0.44	2.10
25% Reduction in low racial diversity communities	15.73	76.35	5.38	0.44	2.11
50% Reduction in low racial diversity communities	11.74	47.08	31.71	6.54	2.94
75% Reduction in low racial diversity communities	10.95	34.95	41.54	9.32	3.24
100% Reduction in low racial diversity communities	15.41	76.69	5.36	0.43	2.12
50% Reduction in Hispanic-major communities	15.22	31.86	36.77	12.38	3.77
100% Reduction in Hispanic-major communities	20.45	37.81	31.55	7.50	2.69
50% Reduction in black-major communities	15.15	54.71	21.16	6.38	2.59
100% Reduction in black-major communities	49.50	34.65	11.88	1.98	1.98
50% Reduction in white-major communities	32.45	60.08	5.30	0.57	1.60
100% Reduction in white-major communities	15.49	67.53	11.36	3.29	2.34
50% Reduction in Asian-major communities	15.22	76.93	5.29	0.40	2.16
100% Reduction in Asian-major communities	15.70	76.37	5.28	0.44	2.11

**Table 14:** Battery: proportion results of false negative on each ethnicity in September–December 2024

Scenario	Hispanic (%)	Black (%)	White (%)	Asian (%)	Other races (%)
0% Reduction	24.12	33.36	31.72	7.73	3.08
25% Reduction in low racial diversity communities	24.62	33.36	31.33	7.63	3.06
50% Reduction in low racial diversity communities	25.59	34.39	29.69	7.32	3.02
75% Reduction in low racial diversity communities	25.96	36.11	28.18	6.79	2.96
100% Reduction in low racial diversity communities	24.69	33.14	31.44	7.67	3.06
50% Reduction in Hispanic-major communities	27.41	35.83	27.51	6.33	2.92
100% Reduction in Hispanic-major communities	29.31	35.21	26.48	6.16	2.85
50% Reduction in black-major communities	24.82	34.82	30.10	7.23	3.03
100% Reduction in black-major communities	22.31	45.75	23.59	5.61	2.74
50% Reduction in white-major communities	24.16	33.32	31.70	7.73	3.09
100% Reduction in white-major communities	24.64	33.67	31.12	7.52	3.05
50% Reduction in Asian-major communities	24.75	32.95	31.62	7.71	3.07
100% Reduction in Asian-major communities	24.61	33.39	31.31	7.63	3.06

**Table 15:** Deceptive practices: proportion results of false positive on each ethnicity in September–December 2024

Scenario	Hispanic (%)	Black (%)	White (%)	Asian (%)	Other races (%)
0% Reduction	23.86	40.18	28.44	4.80	2.71
25% Reduction in low racial diversity communities	26.03	36.18	30.14	4.93	2.71
50% Reduction in low racial diversity communities	30.03	29.38	32.42	5.40	2.77
75% Reduction in low racial diversity communities	27.03	29.62	34.67	5.81	2.87
100% Reduction in low racial diversity communities	28.32	28.16	34.84	5.74	2.94
50% Reduction in Hispanic-major communities	17.96	38.63	34.11	6.18	3.12
100% Reduction in Hispanic-major communities	11.56	45.45	33.99	5.88	3.12
50% Reduction in black-major communities	35.50	23.08	33.13	5.49	2.81
100% Reduction in black-major communities	37.37	13.22	39.80	6.61	3.01
50% Reduction in white-major communities	28.32	50.73	15.48	3.35	2.12
100% Reduction in White-major Communities	27.86	61.21	7.15	2.02	1.76
50% Reduction in Asian-major communities	23.96	38.20	30.15	4.92	2.76
100% Reduction in Asian-major communities	25.29	42.13	25.38	4.62	2.58

**Table 16:** Deceptive practices: proportion results of false negative on each ethnicity in September–December 2024

Scenario	Hispanic (%)	Black (%)	White (%)	Asian (%)	Other races (%)
0% Reduction	33.91	23.42	31.59	7.81	3.28
25% Reduction in low racial diversity communities	30.86	28.54	29.78	7.53	3.28
50% Reduction in low racial diversity communities	26.42	37.62	26.36	6.50	3.11
75% Reduction in low racial diversity communities	28.28	36.70	25.88	6.11	3.03
100% Reduction in low racial diversity communities	28.62	37.99	24.36	6.11	2.91
50% Reduction in Hispanic-major communities	40.14	23.85	26.71	6.42	2.89
100% Reduction in Hispanic-major communities	40.88	23.92	26.28	6.11	2.82
50% Reduction in black-major communities	22.33	43.24	25.18	6.20	3.05
100% Reduction in black-major communities	21.19	53.76	17.85	4.51	2.69
50% Reduction in white-major communities	27.19	18.03	42.28	8.85	3.66
100% Reduction in white-major communities	26.64	17.83	43.21	8.69	3.63
50% Reduction in Asian-major communities	33.71	26.00	29.49	7.56	3.24
100% Reduction in Asian-major communities	31.58	22.21	34.99	7.81	3.41

**Table 17:** Assault: proportion results of false positive on each ethnicity in September–December 2024

Scenario	Hispanic (%)	Black (%)	White (%)	Asian (%)	Other races (%)
0% Reduction	35.03	27.46	27.56	7.33	2.62
25% Reduction in low racial diversity communities	34.84	27.83	27.23	7.46	2.63
50% Reduction in low racial diversity communities	35.90	26.27	27.75	7.50	2.58
75% Reduction in low racial diversity communities	36.62	23.13	29.55	8.08	2.62
100% Reduction in low racial diversity communities	38.62	21.30	29.81	7.69	2.58
50% Reduction in Hispanic-major communities	27.62	33.70	27.21	8.48	2.99
100% Reduction in Hispanic-major communities	23.33	35.80	29.48	8.29	3.10
50% Reduction in black-major communities	38.25	21.95	28.64	8.54	2.61
100% Reduction in black-major communities	44.68	8.79	34.58	9.29	2.65
50% Reduction in white-major communities	37.63	32.68	20.20	7.09	2.40
100% Reduction in white-major communities	39.97	35.01	16.36	6.45	2.22
50% Reduction in Asian-major communities	36.73	27.03	27.19	6.51	2.54
100% Reduction in Asian-major communities	35.41	27.23	28.25	6.51	2.60

**Table 18:** Assault: proportion results of false negative on each ethnicity in September–December 2024

Scenario	Hispanic (%)	Black (%)	White (%)	Asian (%)	Other races (%)
0% Reduction	41.28	18.78	30.32	7.58	2.04
25% Reduction in low racial diversity communities	42.56	17.66	30.54	7.23	2.01
50% Reduction in low racial diversity communities	38.34	23.17	29.24	7.08	2.17
75% Reduction in low racial diversity communities	25.93	51.01	17.60	3.50	1.95
100% Reduction in low racial diversity communities	17.59	64.81	12.44	3.14	2.01
50% Reduction in Hispanic-major communities	55.05	14.69	23.56	5.29	1.41
100% Reduction in Hispanic-major communities	59.43	14.32	19.96	4.54	1.75
50% Reduction in black-major communities	29.87	38.12	25.20	4.52	2.29
100% Reduction in black-major communities	14.83	70.53	9.97	2.36	2.30
50% Reduction in white-major communities	34.45	11.61	44.10	7.13	2.71
100% Reduction in white-major communities	18.53	8.77	59.05	9.73	3.92
50% Reduction in Asian-major communities	36.13	22.13	29.86	9.55	2.33
100% Reduction in Asian-major communities	41.01	18.59	28.45	9.86	2.09

## Footnotes

1 Note that these prediction errors may themselves reflect problems in the input data (such as under-reporting or uneven recording) rather than model behaviour alone.

2 Equivalently, neighbourhood demographics proxy for the exposure of individuals in each ethnic group to police operations.

3 Demographic data on race/ethnicity at the community-area level could not be obtained from the U.S. Census Bureau data because the most relevant source is the 2020 Decennial Census, which does not match our crime-data period (2022–2024).

4 For consistency, these eight categories follow the HAGEN experimental setup for Chicago (Wang et al., 2022).

5 This chronological split follows the same design philosophy as the original HAGEN evaluation (Wang et al., 2022), which also uses a chronological train/validation/test partition. It reflects a realistic deployment scenario in which a model is trained on historical data and applied to future unseen periods. The four-month test period is deliberately longer than in the original HAGEN setup, providing a more demanding evaluation across autumn and winter months.

## Code availability

The original HAGEN code is available athttps://github.com/Rafa-zy/HAGEN/tree/main/HAGEN-code, and the guidance for preprocessing data are available at  https://github.com/sunjiao123sun/CrimeForecaster.git.Any questions on implementation should be directed to the corresponding author.
